# Deep learning–based visual diagnosis of Huanglongbing and zinc deficiency in citrus leaves for sustainable and precision orchard management

**DOI:** 10.3389/fpls.2026.1853541

**Published:** 2026-09-16

**Authors:** Wannaporn Thepbandit, Daniel Martinez Lacasa, Anthikan Klomchit, Dusit Athinuwat

**Affiliations:** 1 Department of Agricultural Technology, Faculty of Science and Technology, Thammasat University, Pathum Thani, Thailand; 2 Department of Innovative Agriculture, College of Creative Agriculture for Society, Srinakharinwirot University, Nakhon-Nayok, Thailand

**Keywords:** Asian citrus psyllid, *Candidatus* Liberibacter asiaticus (*C*Las), Convolutional Neural Networks (CNN), Huanglongbing (HLB), image-based plant disease diagnosis, leaf symptom differentiation, precision agriculture

## Abstract

Citrus greening or Huanglongbing (HLB) is a major threat to global citrus production, yet its visual symptoms often overlap with those of zinc (Zn) deficiency, complicating accurate diagnosis and disease management. This study presents a biologically validated, image-based deep learning method for distinguishing HLB, Zn deficiency, and their co-occurrence in citrus leaves. A total of 2,500 leaves were collected from commercial orchards and imaged using high-resolution flatbed scanning. Five independent expert observers visually classified each leaf into four categories: healthy, HLB, Zn deficiency, and combined HLB and Zn. All samples were subjected to PCR analysis to confirm HLB infection. Comparison between expert assessment and PCR results revealed substantial inter-observer variability and an overall expert diagnostic accuracy of 80.5%, highlighting the inherent limitations of symptom-based visual diagnosis. A hybrid labeling strategy integrating PCR validation for HLB and expert-defined phenotypic evidence for Zn deficiency was used to generate a class-balanced dataset of 1,600 leaves. A multi-label convolutional neural network (CNN) trained on this dataset demonstrated strong diagnostic performance on a PCR-validated validation set, achieving F1-scores of 0.928 (Healthy), 0.936 (HLB), and 0.929 (Zn deficiency), with a Macro-F1 score of 0.931 and an exact-match accuracy of 0.876. Misclassifications were largely confined to physiologically related classes, consistent with overlapping symptom expression. These results demonstrate that deep learning models trained on molecularly validated ground truth can outperform visual diagnosis and serve as scalable decision-support tools for citrus disease management. By enabling more accurate differentiation between HLB infection and nutrient deficiency, this approach can reduce unnecessary or incorrect chemical applications in orchards. Consequently, it supports more precise crop management practices and contributes to sustainable agriculture and environmentally responsible plant protection.

## 1 Introduction

Citrus Huanglongbing (HLB), also known as citrus greening, caused by the phloem-limited bacterium *Candidatus* Liberibacter asiaticus (*C*Las), is widely regarded as the most destructive disease threatening citrus production worldwide (Ghosh et al., 2018; Wang, 2019; Xia et al., 2011). HLB causes severe yield loss, fruit quality decline, premature tree death, and orchard abandonment in major citrus-growing regions (Bassanezi et al., 2020; Djeddour et al., 2021; Pérez-Hedo et al., 2025). The absence of a curative treatment makes early and accurate detection a central component of disease management strategies aimed at limiting pathogen spread and mitigating economic losses.

In commercial orchards, management decisions are frequently guided by visual assessment of foliar symptoms. Typical HLB symptoms include blotchy mottling, asymmetric chlorosis, irregular vein discoloration, and uneven chlorophyll distribution across the leaf lamina (Bové, 2006; Da Graca, 1991). However, these symptoms are notoriously difficult to distinguish from those caused by micronutrient deficiencies, particularly zinc (Zn) deficiency, which is prevalent in many citrus-growing soils. Zinc deficiency disrupts enzyme function and chlorophyll biosynthesis, leading to interveinal chlorosis, reduced leaf expansion, and vein-associated discoloration that can closely resemble early-stage HLB symptoms (Fei et al., 2016; Hambidge and Krebs, 2007). When HLB infection and Zn deficiency occur simultaneously, symptom expression becomes even more complex, producing composite phenotypes that challenge both human interpretation and automated diagnostic systems.

The consequences of misdiagnosis are substantial. When HLB is incorrectly identified as a nutrient disorder, growers often respond with foliar fertilizer applications rather than implementing disease containment measures such as removal of infected trees or intensified vector control. This delay facilitates further pathogen spread by the Asian citrus psyllid while increasing production costs and chemical inputs without addressing the underlying cause (Halbert and Manjunath, 2004). Conversely, mistaking nutrient deficiency for HLB may lead to unnecessary tree removal, resulting in avoidable economic loss. Thus, reliable discrimination between HLB and Zn deficiency is not merely a diagnostic challenge but a critical decision point with direct agronomic and economic implications.

Despite the importance of accurate diagnosis, visual inspection remains inherently subjective. Symptom expression varies with cultivar, tree age, environmental conditions, and disease progression stage, while the uneven distribution of *C*Las within the phloem results in spatially heterogeneous symptoms within and among leaves. Although theoretical distinctions between HLB and Zn deficiency have been described such as asymmetric chlorosis for HLB versus more symmetric patterns for nutrient deficiency, these distinctions frequently break down under field conditions, especially when multiple stresses interact. As a result, even trained inspectors exhibit considerable inter-observer variability and reduced diagnostic accuracy when relying solely on visual cues.

Polymerase chain reaction (PCR)–based assays are widely accepted as the gold standard for HLB detection due to their high sensitivity and specificity (Morán et al., 2023). PCR enables definitive confirmation of *C*Las infection, including in asymptomatic tissues. However, routine PCR screening is costly, labor-intensive, and impractical for large-scale orchard monitoring. Consequently, there is strong interest in complementary diagnostic tools that are rapid, scalable, and capable of operating at the leaf or tree level while retaining biological reliability.

Deep learning has become an established approach for image-based plant disease diagnosis because CNN-based models can learn spatial, color, and texture features directly from leaf images. However, many existing diagnostic models are developed for single-condition classification or visually distinct disease symptoms, whereas practical citrus diagnosis often requires distinguishing between biotic and abiotic stresses with overlapping visual expression.

For HLB diagnosis, two limitations are particularly important: HLB and Zn deficiency can produce similar chlorosis and mottling patterns, and both conditions may occur simultaneously in the same leaf. In addition, datasets based only on visual labels may transfer human diagnostic uncertainty into model training and evaluation. Therefore, a diagnostic framework for this problem should support co-occurring labels and incorporate biologically reliable reference information. PCR confirmation of *C*Las infection provides an objective reference for HLB status, while expert consensus can support phenotypic identification of Zn deficiency symptoms when direct molecular confirmation is not available. In addition, standardized flatbed scanning can reduce non-biological variation caused by lighting, background, camera angle, and leaf orientation, thereby allowing the model to focus on intrinsic leaf symptoms.

In this study, we developed a controlled, PCR-supported image-based diagnostic framework for differentiating HLB, Zn deficiency symptoms, and their co-occurrence in citrus leaves. Citrus leaf samples were collected from commercial orchards in Chiang Rai Province, Thailand, and imaged using a standardized high-resolution flatbed scanning protocol. HLB status was confirmed by real-time PCR targeting *C*Las, whereas Zn deficiency symptoms was assigned using high-confidence expert assessment based on characteristic foliar symptoms. A class-balanced multi-label dataset was then constructed to represent healthy leaves, HLB, Zn deficiency symptoms, and combined HLB + Zn deficiency symptoms. Several CNN-based architectures were evaluated, and EfficientNet-B3 was selected for detailed analysis based on validation performance and inference efficiency. Model performance was assessed using label-wise F1-score, average precision, exact-match accuracy, confusion-matrix analysis, and Grad-CAM visualization. This design allowed us to evaluate whether a multi-label deep-learning framework could distinguish visually overlapping pathological and nutritional stress symptoms under controlled imaging conditions.

## 2 Materials and methods

### 2.1 Leaf collection and human pre-classification

Leaf samples were obtained from commercial citrus orchards distributed across citrus-growing districts in Chiang Rai Province, Thailand. These orchards cultivate predominantly *citrus reticulata* representing the principal citrus taxa grown in northern Thailand. The region was selected due to its long-standing history of citrus greening as well as Huanglongbing (HLB) prevalence and frequent reports of micronutrient imbalances, particularly Zn deficiency, which often confound field diagnosis.

Sampling was conducted during the peak vegetative period to ensure full expression of foliar symptoms. In each orchard, trees were selected using a stratified block design to capture variability in age, canopy vigor, and management practices. From each tree, mature, fully expanded leaves were collected from both sun-exposed and shaded branches to avoid morphological bias introduced by leaf position. Leaves with mechanical damage, insect feeding, or abiotic scorch were excluded to prevent confounding features.

A total of 2500 leaves of all diagnostic category were obtained, yielding four biologically meaningful groups including group1; Healthy leaves, symptom-free leaves exhibiting uniform dark-green pigmentation, intact venation, and no visible mottling or chlorosis. Group2; HLB-suspected leaves, leaves exhibiting classical blotchy mottle, sectorial chlorosis, asymmetric color distribution across the midrib, or irregular patchiness consistent with early- to mid-stage HLB symptomatology. Group3; Zn-deficient leaves, leaves showing interveinal chlorosis with relatively preserved midrib and lateral veins, chlorotic patterns that follow vein architecture, and symptom distributions characteristic of Zn deficiency. Group4; Suspected co-expression leaves, leaves presenting overlapping symptom signatures, including mottling characteristic of HLB coupled with vein-associated chlorosis indicative of Zn deficiency, suggesting potential simultaneous manifestation of physiological and pathological stressors.

Following collection, each leaf was individually placed into labelled airtight bags and transported to the laboratory within two hours to prevent dehydration and post-harvest discoloration. Leaves were cleaned with towel moistened with clean water to remove dust and surface contaminants, then gently flattened using an acrylic sheet covered with a towel to avoid curvature-induced distortion during imaging.

Leaf imaging was performed using a A4 high-resolution flatbed scanner (Canon Scan LiDE 400, Tokyo, Japan). Each leaf was scanned at a fixed optical resolution of 600 dots per inch (dpi), producing uniform, high-fidelity images with consistent illumination and absence of significant shadow artifacts. The adaxial surface was scanned facing down to standardize visible texture and color appearance across samples to capture complete symptom expression, particularly midrib thickening, venation clarity, and subtle chlorotic gradients relevant for distinguishing HLB from nutrient deficiency. At this setting, each pixel corresponds to approximately 42.3 µm × 42.3 µm on the leaf surface. At 600 dpi, one inch is represented by 600 pixels; because one inch equals 25.4 mm, the physical pixel size was approximately 25.4/600 = 0.0423 mm, corresponding to 42.3 µm × 42.3 µm on the leaf surface which was validated by scanning a metric tape. Images were saved in lossless PNG format to preserve fine venation features and color gradients essential for downstream computational analysis.

Human pre-classification was performed prior to any molecular diagnostics. Five independent plant pathology experts, each with more than ten years of experience diagnosing HLB in Southeast Asia, evaluated all scanned images. Their assessments were conducted blindly, with no access to orchard-level metadata. Each leaf was assigned to one of the four predefined categories. Instances in which expert labels disagreed were flagged for arbitration and retained for later quantification of human labeling uncertainty. These disagreement cases served as an important internal benchmark, enabling comparison between human diagnostic ambiguity and CNN performance.

All leaves designated as HLB-suspected or co-expression were subsequently subjected to PCR analysis to determine the definitive presence or absence of *C*Las, the pathogen associated with HLB. The integration of expert visual assessment with PCR-confirmed ground truth provides a biologically rigorous foundation for evaluating the discriminative capacity of the EfficientNet-B3 model.

### 2.2 HLB symptom confirmation and zinc deficiency symptom determination

Molecular confirmation of *C*Las infection was conducted to establish biologically reliable ground-truth labels for all leaf samples used in the study. Midrib tissues were selected for analysis due to their high concentration of phloem elements, where *C*Las preferentially accumulates. One gram of midrib tissue from each leaf was homogenized in liquid nitrogen and subjected to genomic DNA extraction using a modified cetyltrimethylammonium bromide (CTAB) protocol (Clarke, 2009). Detection of *C*Las was carried out using a real-time PCR assay targeting the 16S rDNA gene, following the protocol described by Li et al. (2006) (Li et al., 2006). Integration of molecular diagnostics with expert visual labels enabled quantification of human misclassification, particularly in leaves exhibiting symptoms resembling nutrient deficiencies such as Zn deficiency. This PCR validation ensured that the dataset used for EfficientNet-B3 model development was anchored in unequivocal biological evidence, thereby minimizing the propagation of labeling errors stemming from human subjectivity or latent infection.

Zinc deficiency symptoms was determined based on characteristic foliar symptoms and supported by leaf zinc concentration analysis. Leaf samples were dried, ground, and digested prior to elemental analysis. Zinc concentration was measured using inductively coupled plasma–optical emission spectrometry (ICP-OES; PerkinElmer Optima 8000). The combination of symptom-based assessment and leaf Zn analysis was used to support the assignment of zinc deficiency symptoms labels.

### 2.3 Image dataset and acquisition standardization

All leaf images used in this study were obtained using the standardized scanning protocol described in Section 2.1. This controlled acquisition setup ensured uniform illumination, consistent viewing geometry, and minimal background variability across all samples, thereby reducing non-biological sources of variation in the image dataset.

The resulting image collection was organized into a structured dataset suitable for supervised deep learning. Images were annotated using a multi-label classification scheme with binary indicators assigned independently for each condition. This multi-label approach allowed each sample to receive independent labels for HLB and zinc deficiency symptoms, enabling representation of both single-condition and co-occurring symptom profiles.

The final dataset comprised 1,600 leaf images distributed across four balanced diagnostic categories: Healthy, HLB, zinc deficiency symptoms, and combined HLB and zinc deficiency symptoms, with 400 images per class. To ensure reproducibility and fair comparison across experiments, the same fixed 80:20 split was applied consistently in all experiments. Accordingly, 1,280 images were used for training and 320 images were reserved for validation, corresponding to 320 training and 80 validation images per class. No separate independent test set was used; therefore, model performance was evaluated on the held-out validation set. By decoupling image acquisition from dataset construction and annotation, this design allows the effects of biological symptom expression to be analyzed independently from acquisition-related artifacts, providing a robust foundation for downstream EfficientNet-B3 model training and performance assessment. As typically done in image classification tasks, each scanned leaf image was labeled at the whole-image level based on expert visual assessment and PCR-supported HLB status, resulting in four diagnostic groups: Healthy, HLB, zinc deficiency symptoms, and combined HLB + zinc deficiency symptoms.

### 2.4 Image preprocessing

A custom preprocessing pipeline was implemented in Python using OpenCV to isolate the leaf region and remove background pixels prior to model training. Because flatbed scanning provides a highly controlled imaging environment with uniform illumination and a stable dark background, traditional image-processing operations were sufficient for robust segmentation and did not require a learned segmentation model. Background removal was performed using Hue – Saturation – Value (HSV) color thresholding followed by morphological filtering to eliminate residual artifacts, including minor shadow regions near the leaf margins. Specifically, the binary mask was refined using erosion and dilation operations and morphological opening to suppress small spurious regions and to improve mask smoothness along leaf boundaries. The final mask was applied to retain only leaf pixels for subsequent processing. Input images were resized to a fixed resolution of 512 x 320 pixels (width x height), selected to preserve some degree of the rectangular aspect ratio consistent with the elongated morphology of leaf samples while maintaining sufficient spatial detail for CNN-based feature extraction. No letterbox resizing was applied. Explainable AI analysis Grad-CAM heatmaps were used to visualize the regions influencing CNN decisions, allowing biological interpretation of model features.

### 2.5 Multi-label network architecture

The classification task was formulated as a multi-label binary classification problem rather than a mutually exclusive multi-class problem. Each image was associated with independent binary labels, allowing multiple visual conditions to be present simultaneously within a single sample. This formulation avoids forced exclusivity between overlapping categories and enables the model to learn shared and partially overlapping visual patterns without imposing artificial class boundaries.

Accordingly, all models were designed to output raw logits, with no activation function applied at the output layer. Training optimized a binary cross-entropy loss with logits, allowing independent optimization of each label and facilitating flexible threshold selection during evaluation.

Multiple CNN architectures were evaluated under this formulation. The explored models included lightweight and high-capacity backbones from several families, such as MobileNetV3 (small and large variants) (Howard et al., 2019), MobileNetV4 (Qin et al., 2024), ResNet (He et al., 2016), EfficientNet (B0–B5) (Tan and Le, 2019), EfficientNetV2 (Tan and Le, 2021), ConvNeXt (multiple scales) (Liu et al., 2022), and Ultralytics YOLO-based classification models, specifically YOLO11 and YOLO12 classification variants, including different model sizes (He et al., 2025; Tian et al., 2025). YOLO models were evaluated only as image-classification backbones; no object detection or bounding-box prediction was performed in this study. The final model produced image-level multi-label classification outputs corresponding to Healthy, HLB, and zinc deficiency symptoms. The HLB + zinc deficiency symptoms class was determined when both the HLB and zinc deficiency symptoms outputs were positive for the same image.

For all backbones, the original classification layer was replaced with a linear layer producing logits for the target labels, while the remaining network structure was preserved. No softmax or sigmoid activation was applied within the model forward pass. All models were trained using randomly initialized weights, with no ImageNet or task-specific pretraining applied for the reported experiments since preliminary tests showed a clear disadvantage on using pre-trained weights. A formal component-wise ablation study was not performed. However, a preliminary backbone comparison was conducted using the same preprocessing and multi-label classification setting which results are summarized in **Table 1**.

**Table 1:** Preliminary comparison of candidate backbone architectures for multi-label citrus leaf classification.

Candidate model	Validation loss	Macro-F1
ConvNeXtV2-base	0.2216	0.9046
ConvNeXtV2-pico	0.2464	0.8925
EfficientNet-B2	0.1550	0.9304
EfficientNet-B3	0.1458	0.9349
EfficientNet-B4	0.1514	0.9392
EfficientNet-B5	0.1711	0.9212
EfficientNetV2-S	0.1672	0.9239
MobileNetV4 Conv AA Medium	0.2365	0.9218
MobileNetV4 Hybrid Medium	0.2320	0.9170
YOLO11m-cls	0.1679	0.9317
YOLO12m-cls	0.1963	0.9274

Based on validation performance and inference efficiency, EfficientNet-B3 was selected as the primary architecture for detailed analysis and reporting. This model consistently demonstrated strong performance on the proposed multi-label task while maintaining a favorable balance between classification accuracy and computational throughput. A schematic overview of the proposed workflow is provided in **Figure 1**, showing the complete pipeline from flatbed-scanned leaf image acquisition and preprocessing to EfficientNet-B3 feature extraction and multi-label diagnostic output.

**Figure 1:**
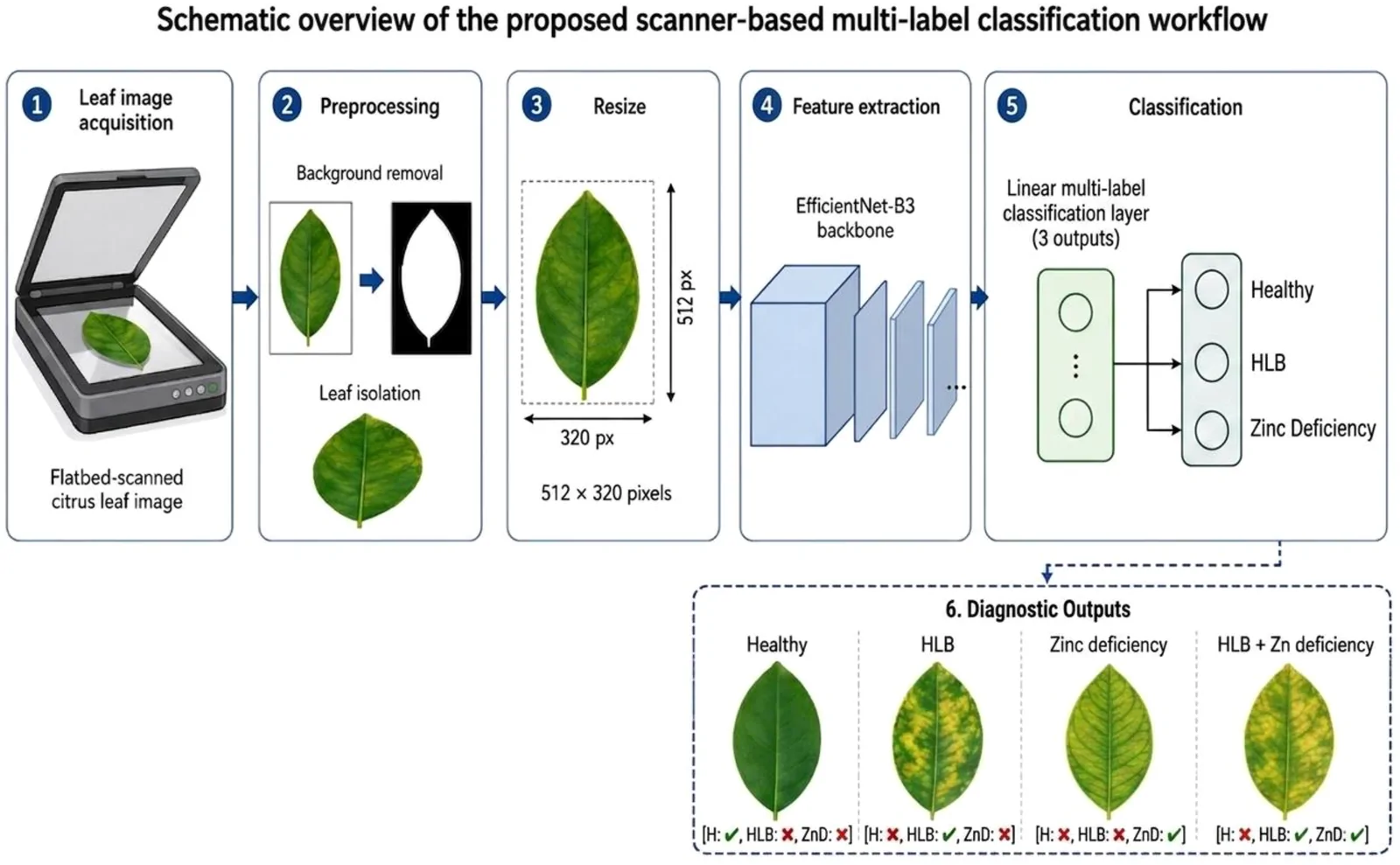
Schematic overview of the proposed scanner-based multi-label classification workflow. Flatbed-scanned citrus leaf images were preprocessed to isolate the leaf region and resized before being passed to the EfficientNet-B3 backbone. The final classification layer produced independent outputs for Healthy, HLB, and zinc deficiency symptoms, allowing single-condition and co-occurring symptom profiles to be represented.

### 2.6 Training hyperparameters and evaluation metrics

All models were implemented in Python using the PyTorch framework. Training was conducted on a workstation equipped with an NVIDIA RTX 5080 16GB and 64GB of RAM under Ubuntu server. Training was performed using the Stochastic Gradient Descent (SGD) optimizer. The training period was set to 150 epochs in which 10 were for warm-up. The learning rate was set to 0.01 following a cosine decay scheduler. The batch size was set to 32. Binary cross-entropy loss with logits was used for all experiments. Multiple data augmentation strategies were applied during training: random horizontal and vertical flipping and color jittering in which brightness, contrast and saturation was randomly altered at a maximum intensity ratio of 0.2. Each experiment was repeated 5 times with different random seeds to assess training stability.

For the model evaluation stage, precision and recall were computed independently for each diagnostic label and combined into an F1-score, which balances false positives and false negatives. A macro F1-score was then calculated as the average of the per-label F1-scores, giving equal importance to all labels regardless of their frequency. Exact-match accuracy was computed as the fraction of samples for which the complete predicted label set exactly matched the ground-truth label set. These metrics were defined as:


$$Precision=\frac{TP}{TP+FP}\;\;\;\;\;\;\;\;\;\;\;\;\;\;\;\;\;\;\;\;\;\;\;\;\;\;\;\;\;Recall=\frac{TP}{TP+FN}$$

$$F_{1}=\frac{2\times Precision\times Recall}{Precision+Recall}\;\;\;\;\;\;\;\;\;\;\;\;\;\;\;\;\;\;\;Macro\;F_{1}=\frac{1}{L}\sum_{l=1}^{L}F_{1,l}$$

$$Exact\;Match\;Accuracy=\frac{1}{N}\sum_{i=1}^{N}1({\hat{\text{y}}}_{i}=y_{i})$$

where TP, FP, and FN represent true positives, false positives, and false negatives, respectively; L is the number of diagnostic labels; N is the number of samples; y_i_ is the ground-truth label vector; ŷ_i_ is the predicted label vector; and 1(·) is the indicator function, equal to 1 when its argument is true and 0 otherwise.

Average Precision (AP) was computed for each label from the corresponding precision–recall curve. AP summarizes how well the model separates positive from negative samples across all possible thresholds and provides a threshold-independent measure of performance. AP values are reported together with the precision–recall curves. Because prediction scores can differ across labels, a single threshold may not be optimal for all labels. Therefore, for each label, the best F1 threshold was determined on the validation set by selecting the threshold that maximized the F1-score. These thresholds help interpret differences between threshold-independent metrics, such as AP, and evaluations performed at a fixed decision threshold. In order to investigate which image regions contributed most strongly to model predictions, Gradient-weighted Class Activation Mapping (Grad-CAM) was applied to trained models. Grad-CAM heatmaps were computed separately for each output logit, allowing independent visualization of feature relevance for each predicted condition. For each sample, gradients were extracted from the final convolutional layer of the backbone network that retained spatial information. Heatmaps were overlaid onto the original input images using a normalized colormap to highlight spatial regions contributing positively to each classification output.

### 2.7 Statistical analysis

All model evaluation metrics were computed using custom Python scripts. Precision, recall, F1-score, Macro-F1, exact-match accuracy, and confusion matrices were calculated from true-positive, false-positive, and false-negative counts, while precision–recall curves and AP were computed using scikit-learn.

## 3 Results

### 3.1 Human pre-classification by expert observers

All leaf samples were initially evaluated through visual inspection by five independent expert observers with experience in citrus disease diagnosis. Each expert independently classified leaves into one of four categories including healthy, HLB, zinc (Zn) deficiency, and combined HLB and Zn based on commonly accepted field diagnostic criteria.

Analysis of expert vote distributions revealed marked differences in inter-observer agreement across symptom classes (**Table 2**). All leaves classified as healthy achieved unanimous agreement among the five experts, resulting in a high-consensus rate of 100%, with no ambiguous cases observed. In contrast, agreement decreased for symptomatic categories. Leaves classified as HLB exhibited a high-consensus rate of 93.12%, while Zn deficiency showed a comparable level of agreement (93.9%). The combined HLB and Zn class displayed the lowest level of expert agreement, with a high-consensus rate of 84.5%.

**Table 2:** Distribution of expert votes (five experts) across symptom classes, stratified by PCR ground truth (N = 2500).

Expert-assigned class	5–0 (full consensus)	4–1	3–2	3–1–1	2–2–1 (no consensus)	Total	High-consensus rate (%)
Healthy	440	0	0	0	0	440	100
HLB	320	492	24	18	17	871	93.12
Zn deficiency	150	328	10	6	15	509	93.9
HLB and Zn deficiency	180	394	46	34	26	680	84.5
Total	1090	1214	80	58	58	2500	—

Five experts independently classified each leaf into one of four classes (Healthy, Huanglongbing (HLB), Zn deficiency, or HLB and Zn deficiency). Vote distributions indicate the number of experts assigning the same class to a given leaf. For example, a 3–1–1 vote pattern denotes that three experts agreed on one class, while the remaining two experts assigned two different alternative classes. Consensus rate was defined as the proportion of leaves achieving majority agreement (≥3/5). The 2–2–1 category indicates no majority consensus and was labeled as ambiguous.

Ambiguous classifications, defined as samples lacking majority agreement (2–2–1 vote distribution), were absent in healthy leaves but present in all symptomatic classes. The proportion of ambiguous cases increased with symptom complexity, occurring most frequently in the combined HLB and Zn category, followed by HLB and Zn deficiency. These results indicate that while expert visual assessment is highly reliable for healthy leaves, diagnostic uncertainty increases substantially when biotic and abiotic stress symptoms overlap.

### 3.2 PCR validation and expert labeling accuracy

PCR validation demonstrated a strong dependence of diagnostic accuracy on the level of expert agreement. Samples with full consensus (5–0) showed the highest concordance with molecular results (92.0%), followed by the 4–1 group (76.1%). In contrast, accuracy declined substantially for lower-consensus categories, reaching 55.0% for 3–2 and falling below 50% for 3–1–1 and no-consensus (2–2–1) samples. Overall, expert visual classification achieved an accuracy of approximately 78–80% relative to PCR, highlighting both the strengths of expert assessment under clear symptom presentation and its limitations in ambiguous cases (**Table 3**).

**Table 3:** PCR validation of expert visual classification stratified by vote consensus level (N = 2500).

Expert vote category	No. of leaves (n)	PCR-confirmed correct	PCR-discordant	Accuracy (%)
5–0 (full consensus)	1090	1003	87	92
4–1	1214	924	290	76.1
3–2	80	44	36	55
3–1–1	58	28	30	48.3
2–2–1 (no consensus)	58	14	44	24.1
Overall	2500	2013	487	80.5

Expert vote categories indicate the distribution of classifications provided independently by five experts. PCR-confirmed correct refers to samples in which expert-based classification was concordant with PCR results for *Candidatus *Liberibacter asiaticus. PCR-discordant indicates disagreement between expert classification and PCR results. Accuracy was calculated as the proportion of PCR-confirmed correct samples within each vote category. The 2–2–1 category represents cases with no majority agreement and was considered diagnostically ambiguous.

Following expert-based visual assessment, molecular filtering was applied to establish biologically consistent training labels. Only samples receiving high-confidence expert agreement (5–0 or 4–1) were considered eligible for inclusion in the training dataset. Final class labels were assigned using a hybrid decision framework integrating expert consensus with PCR results as follows.

Leaves classified as Healthy were required to receive a Healthy label from experts with 5–0 or 4–1 consensus and to be PCR-negative for *C*Las, with no visual of Zn deficiency. Leaves classified as Zn deficiency were required to be PCR-negative for *C*Las and to exhibit either high-confidence Zn-deficiency symptomatology, as determined by expert consensus and plant nutrient deficiency chart. Leaves classified as HLB were required to be PCR-positive for *C*Las and to show no evidence of Zn deficiency. Leaves classified as combined HLB and Zn deficiency were required to be PCR-positive and to exhibit evidence of Zn deficiency based on consistent visual symptoms.

Samples in which expert consensus and PCR results were discordant, for example leaves visually classified as HLB but testing PCR-negative, were excluded from the training pool and retained as a separate discordant dataset for independent analysis and reporting (**Table 4**).

**Table 4:** Criteria for final class assignment and sample selection for model training.

Final training class	Expert consensus requirement	PCR (HLB status)	Evidence of Zn deficiency	Inclusion in training set	Notes
Healthy	5–0	PCR−	None (no visual)	Yes (randomly sampled to 400)	Clear non-stressed control class
Zn deficiency symptom	5–0 or 4–1	PCR−	High-confidence Zn-deficiency symptoms	Yes (randomly sampled to 400)	Abiotic stress without HLB
Huanglongbing (HLB)	5–0 or 4–2	PCR+	None	Yes (randomly sampled to 400)	Biotic stress without Zn deficiency
HLB + Zn deficiency	5–0 or 4–3	PCR+	High-confidence Zn-deficiency symptoms	Yes (randomly sampled to 400)	Combined biotic and abiotic stress
Discordant samples	Any	PCR result inconsistent with expert label	Any	No (excluded from training)	Retained for independent analysis

### 3.3 EfficientNet-B3 classification performance on the validation set

A class-balanced training dataset comprising 1,600 leaf samples (400 per class) was generated through stratified random sampling from high-confidence expert-labeled images, defined as samples receiving 5–0 or 4–1 consensus among the five observers. Prior to sampling, molecular filtering was applied to ensure biological consistency (**Figure 2**).

**Figure 2:**
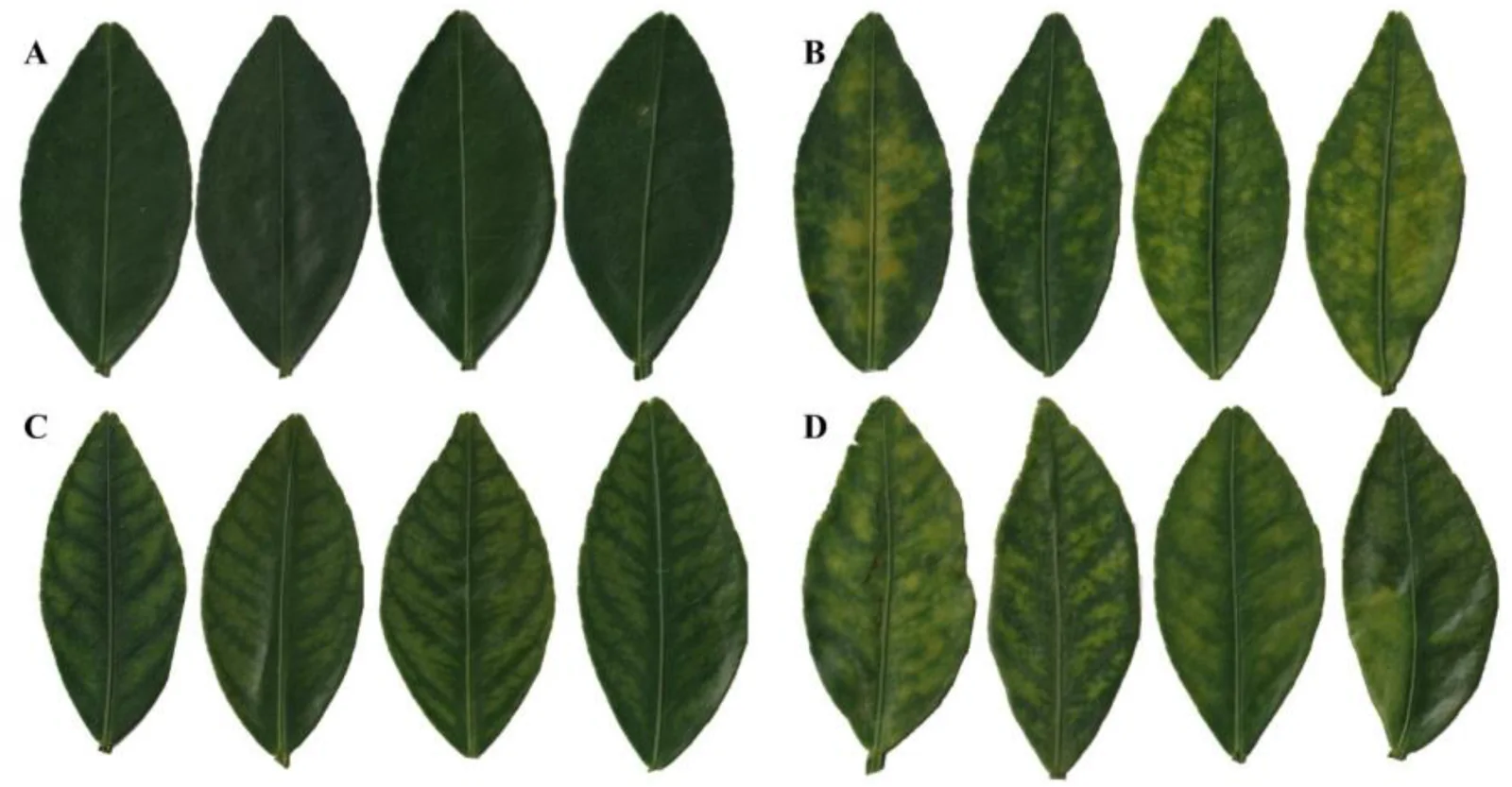
Representative citrus leaf samples and dataset overview used in this study. **(A)** Healthy leaves exhibiting uniform green coloration, intact venation, and absence of visible chlorosis or mottling. **(B)** Citrus greening (Huanglongbing, HLB)–affected leaves showing characteristic irregular, asymmetric blotchy mottling and non-uniform chlorotic patterns not constrained by vein architecture. **(C)** Zinc (Zn)-deficient leaves displaying vein-associated interveinal chlorosis with relatively preserved midrib and lateral veins, resulting in more regular and symmetrical symptom distribution. **(D)** Leaves exhibiting combined HLB and Zn deficiency symptoms, characterized by overlapping features including HLB-like blotchy mottling together with vein-associated chlorosis, reflecting potential co-expression of pathological and nutritional stress.

The performance of the proposed multi-label EfficientNet-B3 classifier was evaluated on the held-out validation set using both label-wise and overall multi-label metrics (**Table 5**). Overall, the model demonstrated strong and consistent performance across all diagnostic categories, indicating effective discrimination between healthy leaves, HLB, and Zn deficiency, as well as robust handling of overlapping symptom expression.

**Table 5:** Summary of multi-label classification performance on the validation set.

Performance type	Metric	Class		
		Healthy	Huanglongbing (HLB)	Zing (Zn) deficiency
Label-wise performance	F1-score	0.928	0.936	0.929
	Average Precision (AP)	0.992	0.985	0.973
	Best F1 threshold	0.6	0.44	0.39
Overall performance	Macro-F1	0.931		
	Exact-match accuracy	0.876		

At the label level, high F1-scores were obtained for all three conditions, with values of 0.928 for Healthy, 0.936 for HLB, and 0.973 for Zn deficiency. These results indicate a balanced trade-off between precision and recall across classes, despite the visual similarity and partial symptom overlap between HLB and Zn deficiency. Average Precision (AP) values further confirmed the strong discriminative capacity of the model, reaching 0.992 for Healthy, 0.985 for HLB, and 0.973 for Zn deficiency, reflecting high ranking quality across a wide range of decision thresholds (**Figure 3**).

**Figure 3:**
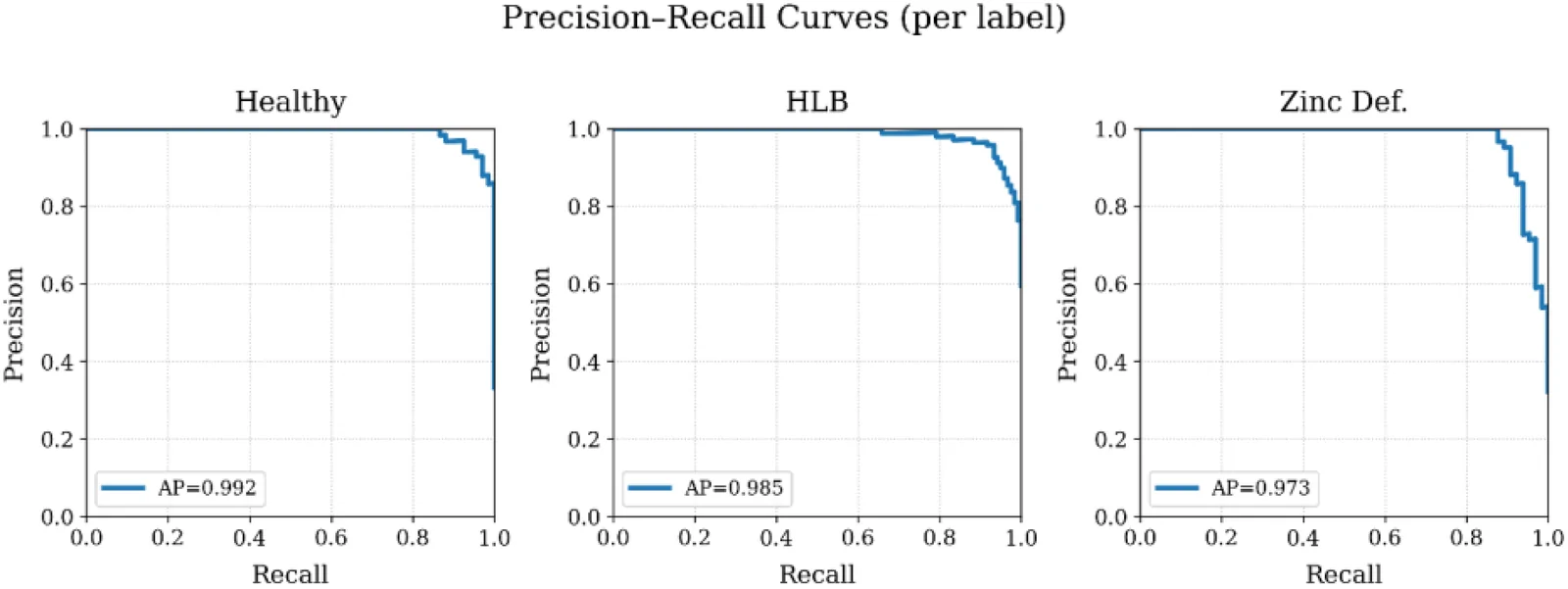
Precision–Recall (PR) curves for each diagnostic label on the validation set including Healthy, Huanglongbing (HLB), and Zinc (Zn) deficiency.

The optimal decision thresholds that maximized the F1-score differed substantially among classes, with best F1 thresholds of 0.60 for Healthy, 0.44 for HLB, and 0.39 for Zn deficiency. This variation highlights the asymmetric confidence distributions across labels and supports the use of class-specific thresholds rather than a single global cutoff in multi-label classification settings.

At the dataset level, the model achieved a Macro-F1 score of 0.931, indicating strong overall performance when treating all labels equally. In addition, an exact-match accuracy of 0.876 was obtained, demonstrating that the model correctly predicted the full label combination for a large majority of validation samples, including cases with overlapping disease and Zn deficiency symptoms. The exact-match accuracy was lower than the label-wise F1-scores because it requires all labels to be predicted correctly. Thus, samples with both HLB and zinc deficiency were marked incorrect when the model identified only one condition.

Taken together, these results demonstrate that the proposed multi-label classification framework can reliably capture both distinct and co-occurring visual symptom patterns in citrus leaves, supporting its suitability for multi-label disease and nutrient stress classification under controlled imaging conditions. In terms of computational efficiency, the selected EfficientNet-B3 model achieved an average inference time of approximately 3 ms per image on the workstation used in this study.

### 3.4 Error structure analysis based on the confusion matrix

The structure of classification errors was examined using the confusion matrix (**Figure 4**) to characterize systematic patterns of misclassification across diagnostic categories. Overall, errors were not randomly distributed but were largely confined to biologically related classes, indicating that misclassifications reflected meaningful visual ambiguity rather than model instability.

**Figure 4:**
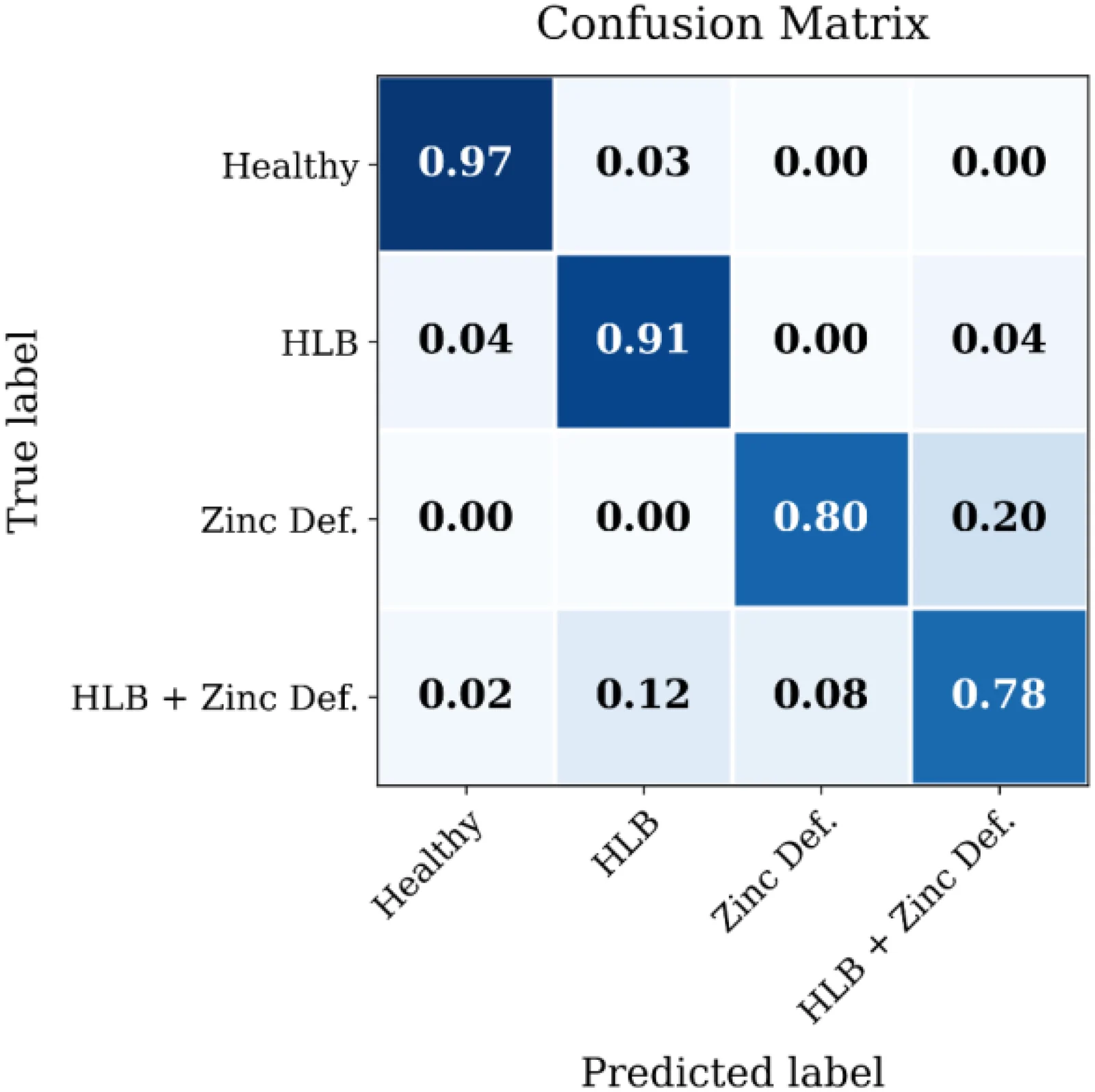
Confusion matrix of classification model [Healthy, Huanglongbing (HLB), and Zinc (Zn) deficiency] at confidence threshold set to 0.5.

Healthy samples were correctly classified in the vast majority of cases (0.97), with only limited confusion toward the HLB class (0.03) and negligible misassignment to other pathological categories. This result suggests that the model reliably captures the visual homogeneity of symptom-free leaves.

Huanglongbing (HLB) samples were predominantly assigned to the correct class (0.91). When misclassification occurred, it was primarily toward the combined HLB and Zn deficiency category (0.04), rather than toward healthy or Zn-only classes. This pattern suggests sensitivity to shared visual cues associated with disease progression and secondary nutritional stress.

Zinc-deficient samples exhibited the highest degree of confusion, with 0.80 correctly classified and 0.20 misassigned to the combined class. Similarly, samples labeled as combined HLB and Zn deficiency were correctly classified at a rate of 0.78, with most errors directed toward the HLB (0.12) and Zn deficiency (0.08) classes. Collectively, these error distributions indicate that misclassifications primarily occur along clinically and physiologically plausible boundaries, consistent with overlapping symptom expression.

These misclassifications likely occurred because the model relied on visual features such as chlorosis, mottling, and vein-associated color patterns. Since HLB and zinc deficiency pattern can produce overlapping symptoms, the model may detect the more visually dominant feature in a leaf while missing the weaker co-occurring feature. This explains why most errors occurred among HLB, zinc deficiency symptoms, and the combined class rather than among unrelated categories.

### 3.5 Performance under pathologically challenging conditions and model interpretability

To further evaluate model robustness under diagnostically challenging scenarios, performance analysis was restricted to samples exhibiting at least one pathological condition including HLB, Zn deficiency, or combined HLB and Zn deficiency. Under this condition, exact-match accuracy decreased to 0.8296, reflecting the increased difficulty of resolving overlapping or borderline visual patterns when multiple stress factors co-occur. Such a reduction is expected in multi-label classification settings and highlights the intrinsic ambiguity associated with compound symptom expression.

Within this pathological subset, Zn deficiency samples remained largely identifiable, with 86.7% correctly classified. Misclassifications (13.3%) were predominantly directed toward the combined class rather than unrelated categories, indicating partial recognition of Zn-associated visual features rather than complete diagnostic failure. This behavior aligns with, but is not redundant to, the confusion matrix analysis, as it isolates performance specifically under high-ambiguity conditions.

To investigate the visual basis of model decisions, Grad-CAM visualizations were generated for representative samples across prediction classes (**Figure 5**). The resulting activation maps demonstrate that EfficientNet-B3 predictions are driven primarily by localized regions within the leaf surface, rather than by global leaf shape or background artifacts. Across classes, salient regions consistently corresponded to internal leaf texture and areas adjacent to venation, features known to be informative for distinguishing pathological and nutritional stress, while background activation remained negligible due to controlled image acquisition and segmentation.

**Figure 5:**
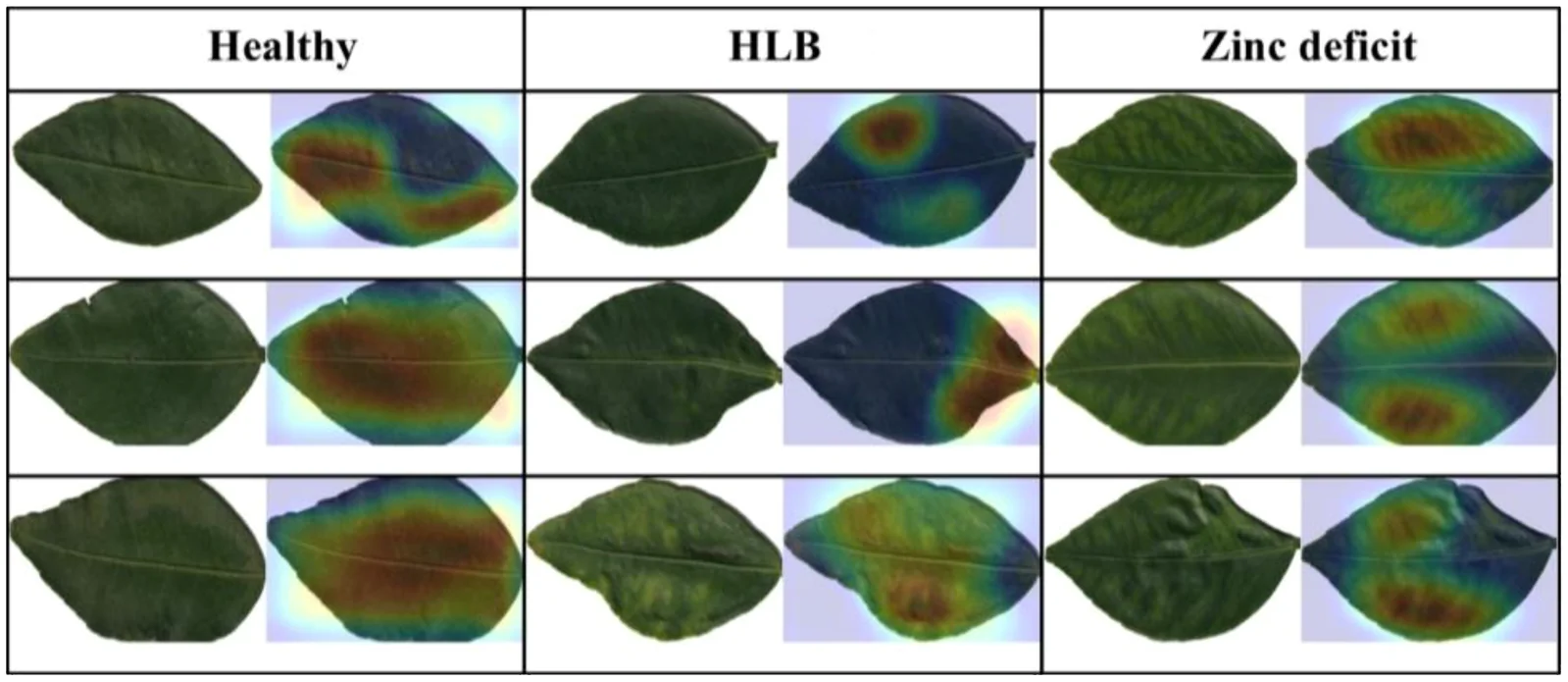
Grad-CAM heatmaps for representative samples across prediction classes including Healthy, Huanglongbing (HLB), and Zinc (Zn) deficiency.

Taken together, these findings indicate that performance degradation under pathological-only evaluation arises from biologically meaningful visual overlap rather than spurious model behavior. The combination of structured error patterns and interpretable attention maps supports the validity of the proposed multi-label framework for citrus leaf symptom classification.

## 4 Discussion

This study focused on HLB, zinc deficiency symptom, and their co-occurrence because these conditions are major diagnostic problems in the citrus production area used as the model site in Chiang Rai Province, Thailand. In this area, HLB and zinc deficiency are commonly observed and may be misdiagnosed because they share similar foliar symptoms, such as chlorosis, mottling, and vein-associated discoloration. Therefore, this study prioritized these two practically important conditions and developed a PCR-supported, image-based multi-label framework to distinguish them and their co-occurrence. Although other citrus disorders may also produce similar symptoms under field conditions, they were not included in the present dataset to maintain a focused diagnostic objective and reliable reference-labeling strategy.

The expert pre-classification results highlight the inherent difficulty of visually diagnosing citrus leaves under conditions of overlapping biotic and abiotic stress.

This finding is consistent with previous reports showing that symptom-based HLB diagnosis is useful but can be unreliable when HLB-like mottling overlaps with nutrient-related chlorosis, particularly under field conditions (Gopal et al., 2010; Tipu et al., 2021). While healthy leaves were identified with perfect inter-observer agreement, both expert consensus and PCR-confirmed accuracy declined progressively as symptom complexity increased. The physiological effects of Zn deficiency in citrus, including altered chlorophyll distribution and vein-associated chlorosis, provide a biological basis for this overlap (Fei et al., 2016). Thus, diagnostic uncertainty is not merely observer-dependent, but reflects intrinsic ambiguity in symptom expression, especially within the combined HLB and Zn deficiency class. The strong dependence of PCR concordance on expert agreement further justifies the exclusion of discordant samples from model training and underscores the necessity of biologically grounded label curation. Collectively, these findings provide a robust rationale for the adoption of a PCR-supported multi-label learning framework, rather than a conventional single-label classification approach, and establish realistic performance expectations for automated diagnosis in this domain.

Building on this foundation, the study demonstrates that high-resolution leaf images acquired via flatbed scanning, when coupled with PCR-validated ground truth, provide a robust and reproducible basis for resolving subtle visual differences between HLB and Zn deficiency. A central implication of this work is that image acquisition methodology plays a critical role in CNN-based classification performance. By minimizing uncontrolled environmental variability related to illumination, background clutter, and viewing geometry, the standardized scanning protocol enables the model to focus on biologically meaningful visual patterns rather than compensating for acquisition-related noise. In this context, flatbed scanning yields a reproducible benchmark dataset that supports fair comparison across model architectures and training strategies, while facilitating detailed analysis of learned representations.

The present framework was developed for standardized high-resolution leaf imaging rather than direct classification of uncontrolled orchard photographs. Flatbed scanning was intentionally used to reduce variation in illumination, background, viewing geometry, leaf orientation, and scale, allowing the model to focus on intrinsic leaf symptoms associated with HLB and zinc deficiency symptoms. Therefore, the reported performance should be interpreted within the scope of a controlled, scanner-based diagnostic workflow.

However, the model has not yet been validated on orchard-acquired images. Field images may include variable lighting, shadows, complex backgrounds, different leaf orientations, and partial occlusion, which can importantly reduce model performance. Future work may evaluate whether the framework can be extended to field-image diagnosis using dedicated field datasets, field-specific augmentation, fine-tuning, or domain-adaptation approaches.

Building on this rationale, another key contribution of this work lies in addressing limitations of class exclusivity assumptions commonly adopted in plant disease classification. Many previous studies formulate disease recognition as a single-label problem, implicitly assuming that each sample belongs to exactly one category. In practice, however, citrus leaves frequently exhibit overlapping visual phenomena arising from co-occurring biotic and abiotic stresses, particularly in the case of HLB and micronutrient deficiencies (Ghosh et al., 2022; Polek et al., 2007; Tipu et al., 2021). The proposed logits-based multi-label image classification method explicitly avoids forced exclusivity and allows independent learning of overlapping visual features. This design choice results in more flexible and interpretable predictions and better reflects biological reality, consistent with recommendations from the broader multi-label learning literature. Evaluation results further highlight the importance of metric selection in multi-label diagnostic settings. Label-wise metrics such as precision–recall curves, Average Precision, and F1-scores assess each label independently and are therefore largely insensitive to cross-label interactions. As a consequence, these metrics can remain high even when the predicted label set for a given sample is partially incorrect. In contrast, exact-match accuracy constitutes a substantially stricter criterion, penalizing any deviation from the full ground-truth label combination. The observed gap between strong label-wise performance and lower exact-match accuracy thus reflects the intrinsic difficulty of multi-label classification under visual correlation, rather than model overconfidence or evaluation bias. Similar discrepancies between label-wise and set-level metrics have been reported in medical imaging and plant stress phenotyping tasks involving correlated conditions (Abdel Maksoud et al., 2019; Gibaja and Ventura, 2015). From a diagnostic perspective, partial correctness may still convey meaningful information. For example, misclassifying a Zn-only sample as a mixed HLB and Zn condition indicates that Zn-related visual features were detected alongside additional patterns associated with another stress factor, consistent with known ambiguity in overlapping symptom expression. Importantly, confusion with the “Healthy” class remained minimal across analyses, suggesting that the model reliably discriminates between healthy and non-healthy samples even under strict evaluation criteria. This behavior mirrors expert diagnostic uncertainty, where disagreement increases primarily among symptomatic categories while healthy tissue remains readily identifiable (Fei et al., 2016; Gopal et al., 2010; Gottwald et al., 2007). Structured error analysis further supports the biological plausibility of model behavior. Misclassifications observed in the confusion matrix were largely confined to physiologically related categories, particularly between Zn deficiency and the combined HLB and Zn class. Such error structure is consistent with the overlapping visual manifestations of nutrient stress and phloem dysfunction and has been interpreted in prior work as evidence that CNNs are learning symptom-relevant representations rather than spurious correlations (Barbedo, 2018).

Interpretability analysis using Grad-CAM provided complementary insight into the visual basis of model predictions. Activation maps consistently emphasized localized regions within the leaf lamina, frequently adjacent to venation and internal texture patterns, while activation outside the leaf area remained negligible. This indicates that model predictions were driven by symptom-relevant tissue regions rather than by global shape cues or residual acquisition artifacts, consistent with prior interpretability studies under controlled imaging conditions (Selvaraju et al., 2017; Toda and Okura, 2019). In the context of HLB, venation-associated activation is biologically plausible given the central role of phloem disruption in symptom development (Pandey et al., 2022). Taken together, these results indicate that no single metric fully captures performance in a multi-label diagnostic setting. Label-wise metrics quantify feature detection capability, whereas exact-match accuracy reflects set-level diagnostic precision. Reporting both provides a more transparent and realistic assessment of model behavior, particularly when classes are visually correlated and co-expression is common.

Overall, this study demonstrates that standardized high-resolution imaging combined with PCR-grounded labels enables EfficientNet-B3 to distinguish visually similar citrus disorders that challenge even experienced human observers, while providing a biologically faithful and methodologically rigorous framework for multi-label plant disease diagnosis.

## 5 Conclusion

This study demonstrates that high-resolution flatbed-scanned citrus leaf images combined with PCR-validated labels provide a reliable and reproducible basis for distinguishing greening disease or Huanglongbing (HLB), zinc (Zn) deficiency, and their co-occurrence. The controlled image acquisition protocol reduces environmental variability, improving classification accuracy and minimizing diagnostic errors by enabling the EfficientNet-B3 to focus on biologically meaningful visual features. The proposed multi-label framework explicitly accounts for overlapping symptom expression, leading to more accurate and interpretable predictions than conventional single-label approaches. By reducing misclassification among visually similar stress conditions, this approach has the potential to alleviate the diagnostic burden on human experts, support more consistent decision-making, and reduce unnecessary chemical interventions arising from incorrect disease diagnosis. This study demonstrates that standardized flatbed-scanned citrus leaf images combined with PCR-validated labels can support reliable differentiation of HLB, zinc deficiency symptoms, and their co-occurrence. The proposed scanner-based workflow reduces environmental variation and supports consistent diagnostic decision-making. Field-image classification was outside the scope of the present study; therefore, future work may explore this extension using dedicated field-image datasets and robustness-improvement strategies.

Nevertheless, the present study has some limitations that should be considered when interpreting the model performance. First, image acquisition was conducted under controlled conditions using flatbed-scanned leaf samples. Although this approach reduced environmental variation and ensured consistent image quality, further validation under real orchard conditions is required. Field-acquired citrus leaf images may include variations in illumination, background complexity, leaf orientation, and partial occlusion, which could affect model performance. Second, the dataset was restricted to *Citrus reticulata* samples collected from a single geographic region, Chiang Rai Province, Thailand. Because HLB and zinc deficiency symptoms may vary among citrus varieties, germplasms, environmental conditions, and production regions, the model may not generalize directly to other citrus types or orchard systems. Future studies should therefore validate and improve the model using independent datasets collected from multiple citrus varieties, such as sweet orange, grapefruit, and lemon, as well as from different geographic regions and real orchard environments. Such work would help assess the practical applicability of the model and support the development of a more robust diagnostic system for commercial orchard use.

## Data Availability

The raw data supporting the conclusions of this article will be made available by the authors, without undue reservation.
